# Hands4U: A multifaceted strategy to implement guideline-based recommendations to prevent hand eczema in health care workers: design of a randomised controlled trial and (cost) effectiveness evaluation

**DOI:** 10.1186/1471-2458-11-669

**Published:** 2011-08-25

**Authors:** Esther WC van der Meer, Cécile RL Boot, Frank HW Jungbauer, Jac JL van der Klink, Thomas Rustemeyer, Pieter Jan Coenraads, Joost W van der Gulden, Johannes R Anema

**Affiliations:** 1 Department of Public and Occupational Health, EMGO Institute for Health and Care Research, VU University Medical Center, Van der Boechorststraat 7, 1081 BT Amsterdam, the Netherlands; 2 Body@Work, Research Center Physical Activity, Work and Health, TNO-VU University Medical Center, Amsterdam, the Netherlands; 3 Department of Public and Occupational Health, Groningen University Medical Center, the Netherlands; 4 Department of Dermatology, VU University Medical Center, Amsterdam, the Netherlands; 5 Dermatology Department, University Medical Center Groningen, University of Groningen, the Netherlands; 6 Department of Primary and Community Care, Centre for Family Medicine, Geriatric care and Public Health, Radboud University Nijmegen Medical Centre, PO Box 9101, 6500 HB Nijmegen, the Netherlands; 7 Research Center for Insurance Medicine AMC-UWV-VU University Medical Center, Amsterdam, the Netherlands

## Abstract

**Background:**

Workers in wet work occupations have a risk for developing hand eczema. Prevention strategies exist, but compliance to the proposed recommendations is poor. Therefore, a multifaceted implementation strategy (MIS) is developed to implement these recommendations to reduce hand eczema among health care workers performing wet work.

**Methods/Design:**

This study is a randomised controlled trial in three university hospitals in the Netherlands. Randomisation to the control or intervention group is performed at department level. The control group receives a leaflet containing the recommendations only. The intervention group receives the MIS which consists of five parts: 1) within a department, a participatory working group is formed to identify problems with the implementation of the recommendations, to find solutions for it and implement these solutions; 2) role models will help their colleagues in performing the desired behaviour; 3) education to all workers will enhance knowledge about (the prevention of) hand eczema; 4) reminders will be placed at the department reminding workers to use the recommendations; 5) workers receive the same leaflet as the control group containing the recommendations. Data are collected by questionnaires at baseline and after 3, 6, 9 and 12 months. The primary outcome measure is self-reported hand eczema. The most important secondary outcome measures are symptoms of hand eczema; actual use of the recommendations; sick leave; work productivity; and health care costs.

Analyses will be performed according to the intention to treat principle. Cost-effectiveness of the MIS will be evaluated from both the societal and the employer's perspective.

**Discussion:**

The prevention of hand eczema is important for the hospital environment. If the MIS has proven to be effective, a major improvement in the health of health care workers can be obtained. Results are expected in 2014.

**Trial registration number:**

NTR2812

## Background

Occupational skin diseases are considered to be in the top-3 of work-related disorders in Europe [1]. Hand eczema accounts for 90% of the occupational skin diseases [2]. One of the most important risk factors for developing hand eczema is exposure to irritants [1]. Water, soap, cleaners, detergents and solvents are among the most common irritants [1,3]. As a consequence, workers who handle these irritants in their work have a higher risk to develop hand eczema [3]. Because of this, especially nurses are at risk for developing hand eczema [3,4]. In a recent study in the Netherlands, the 1-year prevalence of self-reported hand eczema among health care workers, including cleaners, in a university medical hospital was 24% [5]. In comparison, in the general population the 1-year prevalence is nearly 10% [6]. In addition, other occupations in a hospital - like food service workers, and workers in the laboratory [7]- are more often affected by hand eczema than workers in other professions.

Hand eczema is a problem for both the worker facing it and for the society. The physical and psychosocial burden for patients with skin diseases is comparable to patients with other chronic diseases [8]. Cost for society are related to medical consumption [9], sick leave [10] and loss of productivity [11]. Annual costs of medical care, absenteeism and disability pensions due to occupational skin diseases are estimated at € 98,1 million in the Netherlands [12].

However, hand eczema does not only lead to health effects and health care costs in the health care worker who suffers from hand eczema, but it may also have consequences for his or her patients. Several studies report that having hand eczema is a reason for non-compliance to hand hygiene protocols in hospitals [13,14]. Low adherence to hand hygiene procedures causes 20% of health care associated infections [15]. The prevalence of these infections is 6.9%, with a mortality rate of 2% and related costs of €384 million yearly [16,17]. Moreover, Staphylococcus aureus is more frequently present on the hands of patients with hand eczema than persons without it [18]. This poses a risk for workers handling food or working with patients [18]. Consequently, a reduction in the cases of hand eczema among health care workers can lead to a reduction in health care associated infections, mortality and related costs.

The high prevalence of hand eczema among health care workers - nurses, cleaners, food handlers, workers in the laboratory - and the related cost for society indicate that prevention of hand eczema is needed. For this purpose the Dutch Board for Occupational Medicine (NVAB) developed an evidence based guideline to reduce occupational hand eczema [19]. The guideline emphasizes two ways to eliminate and reduce hand eczema. First, through elimination or replacement of the contact factor responsible for the development of hand eczema [19] - in the case of health care workers this is mostly water, soap and wearing occlusive gloves [20] - and second by using preventive measures that support the skin barrier function - e.g. (cotton under) gloves and barrier creams - if avoidance of the contact factor is not possible [21,22].

Despite the problems of hand eczema among health care workers, the implementation of the recommendations from the guideline is poor. There are several reasons for this. First, many nurses - the main group of our study - consider their hand eczema as a standard side effect of their occupation. They hardly notice it anymore [23]. Further, the recommendations advise to use hand alcohol in stead of water and soap for disinfection, though a lot of nurses consider hand alcohol as violent for their skin. As a result, the compliance to this measure is low [24]. Also, a study of Held et al [25] reported difficulties towards the use of individual preventive measures, such as the use of protective gloves and recommended high fat skin care products in wet work occupations - nursing, cleaning and kitchen - in a hospital [23]. For hand hygiene measures it is known that a lack of senior role models is a contributing factor for non-compliance [14].

To implement the recommendations derived from the NVAB-guideline, effective strategies are needed aiming at the organisational and individual level. Multifaceted implementation strategies have shown to be more effective than single strategies [26]. Educational (including reminders [26]) and participatory strategies, like participatory working groups, have shown to be effective for respectively the prevention of skin diseases and musculoskeletal disorders on a small scale [21,25-28]. Further, a systematic review on the effectiveness of prevention programmes for hand dermatitis [29] found that there is moderate evidence for the effectiveness of skin care education including skin protection measures as promoted in the NVAB-guideline. In addition, using trained senior workers as role models, who stimulate the co-workers to use the preventive skin measures, has proven to be effective as an implementation strategy for preventive measures for skin disorders [25].

Although the proposed implementation strategies have proven their effectiveness, there are no studies on hand eczema using senior role models and participatory working groups for health care workers on a large scale. Therefore, a large scale intervention study is needed to evaluate the effects of using multifaceted strategies. The strategies are aimed at the implementation of a prevention program based on the NVAB-guideline. The recommendations in this guideline focus on to reduction of work-related skin disorders among health care workers. The main objective is to evaluate the effectiveness of the multifaceted implementation strategy on the frequency of (episodes of) hand eczema in health care workers. Secondary objectives are: 1) to compare effectiveness of the multifaceted strategy on adherence to the recommendations, general health status of the hands, sick leave and work productivity, and 2) to evaluate the cost-effectiveness of the multifaceted implementation strategy compared to usual practice.

## Methods/Design

### Study design

The Hands4U study is a two-armed randomised controlled trial (RCT). Randomisation is performed at the department level. Workers of the department allocated to the intervention group will receive the multifaceted implementation strategy (MIS); departments allocated to the control group will receive usual practice (no MIS). Data on all outcome measures are assessed at baseline, and 6 and 12 months after baseline. Data on the primary outcome measure (the frequency of (an episode of) eczema on the hands or forearms), as well as on adherence to the recommendations, sick leave, work productivity and health care costs are collected retrospectively every 3 months. The data collection started in April 2011.

The study protocol was approved by the Medical Ethics Committee of the VU University Medical Center. In this study, departments are included as a whole. Therefore, the Medical Ethics Committee decided that participants did not have to sign an informed consent.

### Study population and setting

Participants are health care workers recruited from the departments of three university medical hospitals in the Netherlands. In total 30.000 people are employed at these three hospitals. We only include departments were wet work (e.g. hand washing, wearing gloves) is performed. We estimated that approximately 70% of the workers in the participating hospitals perform wet work.

All departments where wet work is involved are eligible for participation in this study. When a department decides to participate, all workers from the department are invited to participate in the study. Inclusion criteria are: 1) employed at one of the participating hospitals; 2) able to fill out Dutch questionnaires; 3) aged between 18 years and 64 years; and 4) working for at least 8 hours a week. Exclusion criteria is: 1) not performing wet work (e.g. performing administrative tasks).

### Sample size

The one-year prevalence of hand eczema in health care workers in this target group is 24% according to a recent pilot study [5]. Because hand eczema is episodic in nature, repeated outcomes assessments are performed. We assume that a 25% difference in the frequency (of episodes) of hand eczema episodes during the past 3 months (primary outcome measure) is the smallest clinical and societal relevant ratio between both groups [21]. Further, the clustered randomisation on department level is taken into account. An average department is considered to have 50 workers.

Based on the results of a comparable study design [30] an intraclass correlation coefficient (ICC) of 0.73 is estimated. By using the ICC, the power analysis revealed that a sample size of 1200 workers - two groups of 600 workers - is needed to detect a 25% decrease of the frequency (of episodes) of hand eczema among the intervention group compared to the control group [21]. This difference can be detected with a power of 80% and an alpha of 0.05.

When taking into account a drop out rate of 20% a total of 1500 participants have to be recruited for this study. We assume that approximately 50% of the workers will respond to our invitation to participate and will complete the baseline questionnaires. Therefore we will invite 3000 participants in three hospitals to participate in this study.

### Randomisation, stratification and blinding

Randomisation to the intervention group receiving the MIS or the control group takes place on the level of departments to avoid contamination between health care workers receiving the MIS or not. Prestratification was performed based on two criteria, creating four groups: workers have contact with patient (material) versus workers have no contact with patient (material); and high vs. low risk for developing hand eczema, assessed by the occupational physician. Based on the sequence of inclusion, randomisation was performed in strata of two by an independent researcher. For practical reasons the randomization will be performed before baseline measurements. Workers are not informed about the outcome of the randomisation and the design of the study i.e. the existence of the two groups (control and intervention). The department managers are explicitly requested not to inform their workers about this. It is impossible to blind researchers, occupational nurses and department managers for the intervention.

### Control intervention

The workers in the control group receive a leaflet containing the evidence-based recommendations derived from the NVAB-guideline to prevent and reduce hand eczema.

### MIS intervention

The intervention group receives the multifaceted implementation strategy which consist of participatory working groups combined with a role model training and an educational program including reminders. They also receive the same leaflet as the control group.

**Figure 1:**
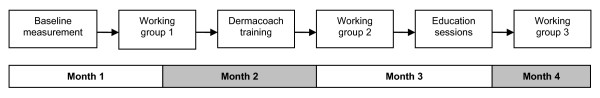
**Time path of the intervention**.

The time path of the intervention can be seen in Figure 1.

#### The participatory working groups

The central part of the intervention are the working groups. The goal of the working group is to identify problems with adherence to the recommendations, to find solutions for these problems and implement these solutions at the department. During three meetings, the working group follows seven steps to accomplish this goal. It is expected that approximately 30 working groups are formed in this study, i.e. 10 working groups per participating hospital.

At each department - consisting of approximately 50 workers - one working group is formed. If a department is larger than 50 workers, more workers are asked to be a part of the working group. The members of the working group are selected by the department manager based on representativeness, their influence on colleagues and their motivation.

Each working group consists of a maximum of eight members. Each member has his/her own task during the meetings. The following persons are included in the working group:

• 3 - 5 representative workers: to identify problems with adherence to the recommendations derived from the NVAB-guideline and to come up with solutions to overcome these problems.

• The manager of the department: to judge whether the developed solutions are realistic and achievable, from both financial and organisational points of view

• A person from the infection control department: to judge whether the solutions are in line with the hand hygiene procedures of the hospital

The working group will be guided by a trained occupational nurse. This occupational nurse received a special training before the start of the study. This training focused on both the theory of (the prevention of) hand eczema as well as on the practical aspects of the intervention.

After a working group is formed the researchers plan three meetings. In case a member of the working group is unable to participate, the department manager selects and asks another worker to participate. If the department manager him/herself is unable to participate, a representative is asked to take his/her place.

The programme of the working group consists of the following seven steps:

#### Step 1 Introduction and inventory of the workplace

At step 1 the working group meets for the first time, approximately one month after the baseline measurement. The trained occupational nurse gives information about the study and about the need for the prevention of hand eczema. After the meeting, the participants and the trained nurse conduct four workplace observations. The goal of these observations is to give the participants insight in their risk behaviour related to hand eczema and to identify possible barriers related to the implementation of the recommendations. The following observations are performed:

• Two participants count how many times they wash their hands, use hand alcohol, use skin care products, and use gloves during the day. They receive a counter for this purpose. Each activity is counted during another day.

• One participant places a flap-over in the lunch room of the department. During a meeting or break the participant asks her colleagues to write down the problems they face with adherence to the recommendations.

• The department manager gives a short questionnaire to key-figures of the department - who do not participate in the working group - containing questions about hand eczema and adherence to the recommendations.

• The trained nurse visits the department and pays attention to adherence to the recommendations, relevant exposures, work organization, wet work, deliverance of work (by others), collaborations with others, instructions, skills, materials and equipment.

The researcher analyses the baseline measurement per department and forms - together with the observations - a document for potential obstacles to adhere/implement the guideline. This document is send to the trained nurse and the participants of the working group before the start of the second meeting.

#### Step 2 Collecting problems with adherence

Under guidance of the trained nurse, the second meeting takes place one month after the first. The meeting starts with a presentation of the document on the workplace observations and baseline characteristics. Problems with adherence to the recommendations are derived from this document and presented to the participants. In addition, the participants brainstorm to collect additional problems with or obstacles to adherence to skin protection measures, using post-its according to the nominal group technique [31]. All mentioned problems/obstacles are prioritized according to severity and frequency of the problem both measured on a 4-point Likert scale (not severe - very severe; not frequent - very frequent). The most frequent and severe problems with adherence are written down on a flap-over. Next, each member can chose three problems he/she finds the most important obstacle for adherence to the guideline by adding a sticker. On the basis of consensus, the three problems with the highest number of stickers are considered to be the three most important obstacles/problems considering adherence to the recommendations.

#### Step 3 Collecting solutions

After the three most important problems are chosen, the working group brainstorms about solutions to overcome these problems using again the nominal group technique. The solutions gathered are prioritized according to criteria of existence, feasibility, complexity and the solving capability of the solution. The manager judges whether the costs of the solutions are feasible; the person from the infection prevention department decides whether the solutions are in line with the hand hygiene procedures. The solutions are prioritized using the same method as in step 2, resulting in a top three of solutions.

#### Step 4 Preparing the implementation

The working group prepares a plan for implementation based on the three most important problems/obstacles and their solutions. The plan describes who is responsible for the implementation of the solutions; what type of activities will be performed by whom, how and when; and whether a test phase is needed. Solutions are divided into either short-term (< 3 months) or long-term solutions (> 3 months). The implementation plan is send to all participants of the working group.

#### Step 5 Implementation of the solutions

The members of the working group implement the solutions, supported by the trained nurse. This implementation phase takes place between meeting two and three. To improve the effectiveness of the implementation process, role models are introduced named Dermacoaches. Dermacoaches are workers from the working group who followed a special training about (the prevention of) hand eczema and are trained on how to promote and enhance the implementation of the solutions and adherence to the guidelines. The Dermacoaches stimulate and motivate their colleagues to be aware of their risk behaviour during work and try to decrease the problems with adherence to the recommendations at the department. They also receive a Dermacoach toolkit which contains posters. These posters are used as reminders to the recommendations for their colleagues at the department and are placed at sinks or other relevant places [26]. Other contents of the toolkit are posters and e-mails with the top-3 problems and solutions prioritized by the working group; and presentations on hand eczema, which they can use during work meetings. Further, the role model assists the trained occupational nurse with the education to the other workers at the department. This is described under the heading *'the education program'*.

#### Step 6 Evaluation of the implemented solutions

A third meeting is planned with the participants of the working group and the trained occupational nurse four to six weeks after meeting two. During this meeting the working group evaluates the implementation of the solutions. Further, the implementation status of the solutions (implemented, not implemented, in progress) is discussed. If needed solutions are adapted to better fit in the organisation.

#### Step 7 Maintenance

The solutions and recommendations will be given a permanent place in the department by using the Dermacoaches, by training new employees in using the recommendations, and by placing the topic on the agenda of meetings regularly during the year.

#### The education program

The goal of the education program is to inform all workers about the risk on hand eczema, the importance of preventive measures and to train them in actual use of individual preventive measures according to the NVAB-guideline, such as the use of hand alcohol, the use of protection measures - such as protective gloves in general and the use of cotton gloves worn underneath rubber and plastic gloves - and the use of recommended high fat skin care products. Most important is that the workers become aware of their own risk behaviour in relation to hand eczema and how to reduce this risk. They also receive information from the Dermacoach about the problems identified by the working group and the solutions for these problems.

The program is a short session (20 minutes) and is planned during a regular meeting of the workers at the department. The program is planned and carried out by the trained occupational nurse and the Dermacoach. More sessions are held to increase the reach of the education.

All workers participating at the session receive a bag containing products related to the prevention of hand eczema, like moisturizers and cotton under gloves. Afterwards, the Dermacoach will place key messages (reminders) at sinks or other relevant places at the department.

### Use of co-interventions

To our knowledge no specific program to prevent hand eczema are performed in the participating departments. However, co-interventions will be assessed in the questionnaire. In addition, department managers are asked whether there are other ongoing studies, planned re-organizations or other relevant changes at the department that can influence implementation. The necessity of knowing whether co-interventions took place is that the control group ideally does not receive any interventions during trial period and the intervention group does not receive any additional interventions. When this does occur the effect of the intervention can respectively fade or increase compared to the control group.

### Goal of the data collection

Eventually, this study will evaluate the procedures in two ways. The first is by performing a (cost-) effectiveness study relating the intervention to the prevention of hand eczema and, secondary, to the use of the recommendations. Considering this as an implementation study, it is important to evaluate the process as well. Therefore, a quantitative process evaluation is performed and in addition a qualitative exploration of barriers and facilitators related to the implementation of the recommendations.

### Data collection procedure

Departments can choose whether they want to receive the questionnaires online or by hard copy. The online questionnaires are sent to the workers by e-mail containing a link to the questionnaire. The hard copy questionnaires are sent to the department managers, who hand out the questionnaires to the workers. The researchers collect the completed questionnaires. Approximately one month before the start of the working groups the baseline questionnaire is sent to the workers. In order to enhance the response a maximum of three reminders will be send. Further, the department managers will be asked to encourage their workers to fill out the questionnaires. Subsequently, the researchers visit the departments before, during and after each measurement to encourage workers to fill out the questionnaires. In addition, incentives are used. This procedure has resulted in a inclusion rate of 60% and 20% dropout rate after inclusion in a comparable study to prevent back and neck pain in the target group in the VU University Medical Center [32].

### Outcome measures

#### Primary outcome measure

Primary outcome is defined as the frequency of (an episode of) eczema on hands or forearms within the past three months based on questions D1 ('Have you ever had hand eczema?'), D2 ('Have you ever had eczema on your wrists or forearms?'), and D5 ('When did you last have eczema on your hands, wrists and forearms?') in the Nordic Occupational Skin Questionnaire (NOSQ-2002) [33,34]. Question D5 is measured on a four-point scale containing the options: 'I have it just now', 'Not just now, but within the past 3 months', 'Between 3 - 12 months ago' and 'More than 12 months ago'. A dichotomous variable is created using these three questions for the prevalence of hand eczema in the past three months. An interval of three months is used, because hand eczema has an episodic and recurrent course.

#### Secondary outcome measures

The three questions from NOSQ-2002 only measure the presence or absence of hand eczema, without taking into account the symptoms related to it [33,34]. Therefore, a symptom-based questionnaire is added to measure the symptoms of hand eczema every three months [19].

The impact of hand eczema on the worker's disease-specific quality of life is measured using the DLQI [35]: a 10-item scale which measures the impact of skin diseases on several physical, psychological and social aspects of daily life. For this study we use the Dutch version of the DLQI adapted by Evers et al. [36]. It will be measured at baseline, after 6 and 12 months.

Workers' global assessment of the health of their hands and patients' global assessment of eczema will be assessed at baseline and after 6 and 12 months, using a 11-point Likert scale based on the NOSQ-2002 (question D12) [33].

Skin exposure during work time is measured using an adopted version of the NOSQ-2002 [33,34]. It will be measured at baseline and after 6 and 12 months.

The actual use of preventive measures is measured with a modified version of the NOSQ-2002. The modifications are necessary, because questions have to be in accordance with the specific work environment of the workers. Further, the researchers monitor whether the preventive measures are used or not. In addition to self reported use, information is collected at the department level about the actual use of protective measures like gloves, use of disinfectants and use of barrier creams. This information is gathered by the purchasing department and the pharmacy of the participating hospitals. Both the questions from the NOSQ-2002 and purchase data will be collected every 3 months.

Sick leave is measured by using the PRODISQ [37]: one general question on sick leave and three questions about sick leave due to hand eczema. Work productivity is measured using a single item question from the WHO Health Productivity Questionnaire [38,39] and three single item questions from PRODISQ [37]. All but one question on work productivity are measured on a 11-point Likert scale. Health care costs related to hand eczema are collected with respect to the economic evaluation. All these questions will be assessed every 3 months.

Several questions about the knowledge of prevention of hand eczema are included. Three questions are included asking whether the workers receive information about the prevention of hand eczema and what kind of information (e.g. leaflets, presentation) they receive. This is measured at baseline, after 6 and 12 months.

Psychosocial work characteristics will be measured at baseline and after 6 and 12 months by means of a Dutch version of Karasek's Job Content Questionnaire, containing the following constructs with a reliability between 0.65 and 0.83: decision authority and co-worker support [40]. Because the working groups in this study have to decide what measures they want to implement, it is important to know if there are possibilities for workers to take their own decisions. Therefore, we measure decision authority. Co-worker support is important, because we expect that colleagues have an influence on compliance to the recommendations.

It is known that the actual use of preventive measures for the prevention of musculoskeletal disorders is positively and significantly associated with behavioural change phases [41]. Therefore, the behavioural determinants attitude-social influence- self-efficacy (ASE) [42] for (the intention to perform) the desired behaviour - actual use of preventive measures - are asked using eight questions. This questions will be assessed at baseline, after 6 and 12 months.

#### Prognostic factors

At baseline sociodemographic data (i.e. age, gender, education, working hours per day, working days per week, nationality, job description) are assessed.

Several relevant questions on eczema are assessed at baseline, like eczema in the past - derived from the NOSQ-2002 [33,34] - and atopy [19]. Skin exposure during leisure time is measured on a Visual Analogue Scale (VAS). Questions on exposure in leisure time will be measured at baseline, after 6 and 12 months.

### Statistical analyses

All analyses are performed according to the intention to treat principle. Most analyses are performed at worker level. Two analyses are performed: 1) a crude analysis with the outcome variable measured at follow-up as the dependent variable adjusted for the outcome, measured at baseline, and 2) an analysis as above but adjusted for potential covariates (e.g. gender, age, type of work, history of hand eczema, and skin exposure). Effects of the intervention will be checked for effect modification (gender, type of exposure, number of preventive skin measures implemented). Generalised estimation equations (GEE) are used to analyse long-term results (i.e. 12 months after baseline). Furthermore, analyses at department level are performed by the use of multilevel analysis.

For all analyses a two-tailed significance level of < 0.05 is considered statistically significant. The multilevel statistical analyses are performed with MlwiN 2.0; linear and logistic regression analyses are performed with SPSS 15.0 (SPSS Inc. Chicago, Illinois, USA), and GEE analyses is performed with STATA version 7.0 (College Station, TX).

Considering the episodic nature of hand eczema, the use of transition models is necessary. In these models, the presence of hand eczema in the past three months is incorporated. The probability of getting hand eczema in workers with no hand eczema and the probability of getting no hand eczema in workers with hand eczema are modelled simultaneously by means of a logistic mixed model as is done in a study with a comparative design on low back and neck pain [32]. Transitions models will be conducted using the gllamm procedure in Stata version 10.0.

### Economic evaluation

Cost-effectiveness of the multifaceted implementation strategy will be evaluated from both the societal perspective and the employers' perspective. Both direct and indirect costs will be measured and valued. Indirect costs are not related to health care, but are costs in paid and unpaid labour as a consequence of sickness, sick leave, disability and/or death of a productive person. The indirect costs for production losses due to sick leave are calculated by using the Human Capital and Friction costs method [43]. For the latter method, the Dutch guidelines for economic evaluation is used [44]. The direct health care costs are calculated by using tariffs for the costs of health care professionals and market prices for the value of medication. Costs for the education, role model training and working groups are calculated by using the hourly wages. The direct non-health care costs, are calculated by using the information obtained from the cost questionnaires and shadow prices.

Bootstrapping [45] is used for comparison of mean direct, indirect and total costs between the two groups. Confidence intervals are obtained by bias corrected and accelerated bootstrapping. Cost-effectiveness ratios are calculated by dividing the difference between the mean costs of the interventions by the difference between the mean effects of the interventions. The bootstrapped costs effects pairs are graphically presented on a cost-effectiveness plane. Acceptability curves are calculated in order to show that the probability of the intervention is cost-effective at a specific ceiling ratio. Furthermore, sensitivity analyses are performed.

### Process evaluation

The process of the MIS is evaluated in five ways.

First, the working group is asked for their opinions on 1) the content and process of the working group meeting as a whole, 2) the specialised occupational nurse's competence, 3) their expectations towards the implementation and the effectiveness of the prioritised measures on the adherence to the recommendations.

Second, Dermacoaches are asked for their opinions about 1) the Dermacoach training as a whole, 2) the added value of the training as a role model to improve the implementation process and to improve the actual use of preventive measures, 3) their experiences/perceived effectiveness of being a role model.

Third, all workers of the intervention departments are asked about satisfaction, barriers and facilitators regarding the educational program and if they are aware of prioritised organisational measures and whether these measures are implemented at the department. Further they are asked whether they received the leaflet containing the recommendations for the prevention of hand eczema and their opinion on this leaflet.

Fourth, all members of the working group receive a questionnaire with the following questions: 1) whether the prioritised organisational measure(s) are implemented, 2) to identify and describe possible barriers and facilitators during the implementation of the measure(s). One worker of the working group is invited for a semi-structured interview in which the implementation process is discussed. The content and structure of the interview is based on the answers given in the questionnaires of all working group members.

Fifth, the department manager is also sent a questionnaire and is also invited for a semi-structured interview. The department manager is asked: 1) whether the prioritised organisational measure(s) are implemented, 2) to identify and describe possible barriers or facilitators during the implementation of the measure(s).

## Discussion

Hand eczema is a problem in the health care setting, because of the high prevalence among the workers in this occupational setting [5]. The problems are related to the burden for the individual worker, the employer and the society [8,10-14]. In this study, the effectiveness of a combination of several strategies for the prevention of hand eczema among health care workers will be investigated.

### Theoretical framework

Fleuren et al [46] developed a framework for innovation processes. According to this framework implementation can be affected by four constructs: characteristics of the socio-political context, the organization, the adopting person, and the innovation. In total, 50 determinants are identified to fit in one of these constructs. Depending on the nature of the determinant, the influence can be facilitating or impeding. The innovation strategy, which in this study is the MIS, can influence these determinants. Each aspect of the MIS targets one or more of the determinants indentified by Fleuren et al [46].

First, the socio-political context is taken into account by using the working groups. The working group forms solutions to overcome problems related to the implementation of the recommendations. The socio-political context can be a barrier for implementing the solutions. The working groups takes the socio-political context into account and adapts the solutions to this context. In that way implementation can be enhanced, making sure the socio-political context is not a barrier for implementation.

The working group also plays a part in influencing the characteristics of the organization. A participatory approach in decision making is a facilitating factor for implementation [46]. This participatory working group is formed out of members of the organization, e.g. the department of a hospital. They make sure that the solutions for the barriers to implementation do not conflict with the culture of the organization. Further, the education sessions will heighten the available expertise in relation to the innovation. Another determinant of the organization our intervention could have an influence on is on the presence of an opinion leader. This person influences opinions of others in the organization. The Dermacoaches will influence their colleagues to change their opinion towards the recommendations in a positive way.

The characteristics of the user are influenced by almost all parts of the MIS. The role models give support to their colleagues to use the recommendations and they perform the desired behaviour. In a study on hand hygiene compliance, the latter have shown to be important for enhancing adherence [14]. The education provides the workers with knowledge and skills needed to use the recommendations. The perceived ownership on the implementation of the recommendations is heightened by the working group. Further, the attitude of the workers is influenced by the education sessions.

Finally, the working group is important when considering the characteristics of the innovation. It is important that users, in this case the health care workers, are involved in the development of the innovation. Further, the working group makes sure that the solutions are compatible with the existing work procedures. Education to the workers can enhance the clarity of the recommendations, otherwise the lack of knowledge can form a barrier to the use of the recommendations.

### Methodological considerations

A weakness of this is study is the risk for underreporting. Hand eczema is not a disorder that is considered to be a problem by health care workers, because many consider their hand eczema as a part of their job and are therefore not alarmed by it [23]. The workers who do recognize the problem of their hand eczema may, as a result, not report their eczema. It could therefore be difficult to measure a change in hand eczema in general and between the intervention and control group. Considering the large number of participants in this study, and the high work load of participants, we chose to optimise feasibility by measuring hand eczema by self report rather than an examination of the hands by a physician. A second weakness may be the forming of the working group. Ideally, the workers participating in the working group are role models at the department and are an example to their colleagues. However, we can only partly control the selection of workers and even then the right workers can refuse, leaving the department with a suboptimal composition of the working group. Third, randomisation was performed at department level to avoid contamination, but it is not possible to rule out contamination completely since intervention and control departments will be located in the same hospital. However, participants are not informed about the design of the study i.e. they do not know of the existence of a control and intervention group. In that way the contamination is minimized. That this study focuses not only on primary, but also on secondary prevention, can be considered as a fourth weakness. Workers with symptoms are probably more likely to comply with the recommendations, than workers with no symptoms at all. As a result, the effect of the intervention can be different for the two types of workers.

A first strength of this study is that it is performed in a real world setting. This is not an efficacy study and the study does not only give insight in effectiveness, but also on feasibility. In other words, the effect will come close to the effect in real practice and to the real process of implementation. Second, the intervention is based on previous successful studies in other settings and populations. A systematic review found that education is a promising tool for the prevention of hand eczema [29]. However, the studies using education for primary prevention of hand eczema in the health care setting use a small population [23,25] leaving room for testing the effectiveness of education in a large study population as in our study. Third, this study does not only focus on investigating the effects of the intervention on the prevention of hand eczema, but also on the implementation by studying the actual use of the recommendations for the prevention of hand eczema. Kütting [47] mentioned in his article that in studies on hand eczema only the reduction of hand eczema is taking into account and not the actual use of the recommended products in the intervention. A fourth strength is that working groups are formed in the intervention group to overcome problems with adherence to the recommendations for the prevention of hand eczema. Knowledge does not imply that behaviour will change [48]. Therefore, it is important to focus an intervention on the barriers and problems related to the implementation [48]. This study follows that direction in using the working groups who identify problems with adherence to the recommendations. In the former section, the combining of primary and secondary prevention was considered to be a weakness of the study. In contrast, it is also a strength. Including both healthy workers and workers with hand eczema means that the results of the study can be generalizable to a large group of workers. A final strength is that this study is the first study which combines education, working groups, reminders and role models for the prevention of hand eczema in a health care setting.

### Impact of the results

The prevention of hand eczema is important for the hospital environment. If the intervention used in this study is proved to be effective, a major improvement in the health of health care workers can be obtained.

The intervention offers a ready-to-work with method for the prevention of hand eczema. When proven effective, it can be implemented in other (health care) settings, preventing hand eczema in wet work occupations in the Netherlands.

## Competing interests

The authors declare that they have no competing interests.

## Authors' contributions

All authors contributed to the design of the article and the writing of this paper. They all approved the final manuscript. EWCM is the principle researcher and is responsible for the data collection. CRLB coordinates the study. CRLB, PJC, JWG and JRA supervise the study.

## Pre-publication history

The pre-publication history for this paper can be accessed here:

http://www.biomedcentral.com/1471-2458/11/669/prepub
